# A Rapid Bacteriophage DNA Extraction Method

**DOI:** 10.3390/mps1030027

**Published:** 2018-07-27

**Authors:** Džiuginta Jakočiūnė, Arshnee Moodley

**Affiliations:** Department of Veterinary and Animal Sciences, Faculty of Health and Medical Sciences, University of Copenhagen, 1870 Frederiksberg C, Denmark; djak@sund.ku.dk

**Keywords:** phage, fast, DNA extraction, spin column, genome, sequencing

## Abstract

Bacteriophages (phages) are intensely investigated as non-antibiotic alternatives to circumvent antibiotic resistance development as well as last resort therapeutic options against antibiotic resistant bacteria. As part of gaining a better understanding of phages and to determine if phages harbor putative virulence factors, whole genome sequencing is used, for which good quality phage DNA is needed. Traditional phage DNA extraction methods are tedious and time consuming, requiring specialized equipment e.g., an ultra-centrifuge. Here, we describe a quick and simple method (under four hours) to extract DNA from double stranded DNA (dsDNA) phages at titers above 1.0 × 10^10^ plaque-forming units (PFU)/mL. This DNA was suitable for library preparation using the Nextera XT kit and sequencing on the Illumina MiSeq platform.

## 1. Introduction

Bacteriophages (phages) are of great interest because of their ability to specifically attack bacteria. Phages are approved to be used in the food industry to control foodborne pathogens [1]. Moreover, animal and human clinical trials have demonstrated that phages can be efficiently used as alternative to antibiotics [2], including treatment of infections caused by antibiotic resistant bacteria. However, in order to ensure the safety of the phages, i.e., phages do not harbor putative virulence factors or antibiotic resistance genes that could be transferred to bacteria, whole genome analyses are performed [2], requiring extraction of phage DNA. Traditional extraction methods include precipitation of phages, ultra-centrifugation, the use of hazardous solutions, e.g., chloroform, phenol [3], and are time consuming, e.g., the protocol described in Sambrook takes two days [4]. While viral DNA extraction kits based on spin column technology are commercially available, we describe a simple and fast method using the spin column based DNeasy Blood & Tissue Kit (Qiagen, Hilden, Germany), which is used to extract DNA from blood, tissues, bacterial lysates, insects, and yeast. We chose this kit since it is our preferred in-house kit for extraction of bacterial DNA for whole genome sequencing. We applied a modified protocol to extract DNA from streptococcal phages.

## 2. Experimental Design

Required materials: Brain Heart Infusion Broth (BHI, Oxoid, Basingstoke, UK), Agarose (Merck KGaA, Darmstadt, Germany), CaCl_2_ (Merck KGaA, Darmstadt, Germany), SM buffer [5], DNase I (1 U/µL, Thermo Scientific, Roskilde, Denmark), RNase A (10 mg/mL, Thermo Scientific, Roskilde, Denmark), Proteinase K (3.0–15.0 U/mg solid, lyophilized powder, Merck KGaA, Darmstadt, Germany), 0.5 M ethylenediaminetetraacetic acid (EDTA), DNeasy Blood & Tissue Kit (Qiagen, Hilden, Germany), and 99% ethanol, GeneRuler 1 kb DNA Ladder (250–10,000 bp, Thermo Scientific, Roskilde, Denmark).

Required equipment: Heating block, benchtop centrifuge, shaker, pipettes, filter tips 1 µL–1000 µL, 1.5 mL and 2 mL sterile microcentrifuge tubes, 13 mL centrifuge tubes, 0.22 µm sterile syringe filters, NanoDrop™ (Thermo Scientific, Roskilde, Denmark), Qubit^®^ fluorometer (Thermo Scientific, Roskilde, Denmark).

## 3. Procedure

### 3.1. Propagation of Streptococcal Phages by the Plate Method. Time for Completion: 2–3 days

For propagation of our phages, we used a double agarose overlay plaque assay as previously described [5] with some modifications. It should be noted that this method may not apply to all phages or bacteria, hence users should use their preferred method of phage propagation.

(a) To prepare the bottom layer, BHI broth containing a final concentration of 0.8% agarose is heated to melt the agarose, and then cooled to 56 °C before adding CaCl_2_ to a final concentration of 5 mM. Twenty-five milliliters are dispensed to sterile petri dishes. Solidified plates are stored at 4 °C until use.

(b) To prepare the top layer, BHI broth containing a final concentration of 0.4% agarose is heated to melt the agarose, and then cooled to 56 °C before adding CaCl_2_ to a final concentration of 5 mM.

(c) Prepare SM buffer containing gelatin as described previously [5].

1. Day 1: Determine which dilution of the phage lysate that will produce confluent lysis. Mix 100 µL of this dilution with 4 mL of molten top layer containing 80 µL of an overnight broth culture of the propagation strain. This mix is then poured over the bottom layer. Once the agarose had solidified, plates are incubated upside down at 37 °C overnight.
2. Day 2: Add 5 mL of SM buffer to the plate and incubate at 4 °C overnight with shaking (50 rpm) to elute phages from the top layer.
3. Day 3. Recover the eluted phage by removing the SM buffer to 13 mL centrifuge tubes. Centrifuge at 5 min at 5000× *g* to remove residual bacteria and filter lysate with a 0.22 µm syringe filter.

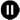
 **PAUSE STEP** Lysates can be kept at 4 °C until further use.

### 3.2. DNA Extraction. Time for Completion: 4 Hours

#### 3.2.1. Removal of Bacterial DNA and RNA, and Digestion of Phage Capsid [4]. Time for Completion: 3 Hours

- To remove any residual bacterial DNA and RNA present in the lysate, 450 µL of the filter-sterilized lysate is incubated with 50 µL DNase I 10x buffer, 1 µL DNase I (1 U/µL), and 1 µL RNase A (10 mg/mL) for 1.5 h at 37 °C without shaking. Thereafter, 20 µL of 0.5 M EDTA (final concentration 20 mM) is added to inactivate DNase I and RNase A.
- To digest the phage protein capsid, 1.25 µL Proteinase K (20 mg/mL) is then added and incubated for 1.5 h at 56 °C without shaking.

#### 3.2.2. DNA Purification with the DNeasy Blood & Tissue Kit (Qiagen, [6]). Time for Completion: 1 Hour for 12 Samples)

1. Add 200 µL of lysed phage lysate to 200 µL of AL buffer, vortex to mix thoroughly and incubate for 10 min at 70 °C. Optional: to achieve a higher DNA yield, a larger volume of the lysed phage lysate can be used and add the equivalent volume of AL buffer.
2. Add 200 µL of 99.9% EtOH (or the equivalent volume if using a larger volume), vortex to mix thoroughly.
3. Transfer the mixture to the DNeasy Mini spin column placed in a 2 mL collection tube (provided with the kit) and centrifuge for 1 min at 6000× *g*. If larger volumes are used, repeat this step several times. Do not load more than 750 µL at a time onto the column. Discard the flow-through and the collection tube.
4. Place the DNeasy Mini spin column in a new 2 mL collection tube (provided), add 500 µL Buffer AW1, and centrifuge for 1 min at 6000× *g*. Discard the flow-through and the collection tube.
5. Place the DNeasy Mini spin column in a new 2 mL collection tube (provided), add 500 µL Buffer AW2, and centrifuge for 3 min at 20,000× *g* to dry the DNeasy membrane. Discard the flow-through and the collection tube. Place the DNeasy Mini spin column in a new 2 mL collection tube (provided) and centrifuge for an additional 1 min at 20,000× *g* to make sure there is no carryover of ethanol.
6. Place the DNeasy Mini spin column in a sterile 1.5 mL or 2 mL microcentrifuge tube (not provided in the kit), and pipette 30 µL AE Buffer directly onto the DNeasy membrane.
7. Incubate at room temperature for 1 min, and then centrifuge for 1 min at 6000× *g* to elute the DNA.
  **OPTIONAL STEP** Repeat the elution step using the eluate from the first elution.

The concentration and quality of the DNA was assessed using the Nanodrop and Qubit according to the manufacturer’s instructions. Five microliters of DNA was also run on a 1% agarose gel. Preparation of the sequencing libraries was done according to Kot et al*.* [7] and sequenced on the Illumina MiSeq platform [8] using MiSeq Reagent Kit v2 (500-cycles) and manufacturer’s instructions.

## 4. Expected Results

**Figure 1:**
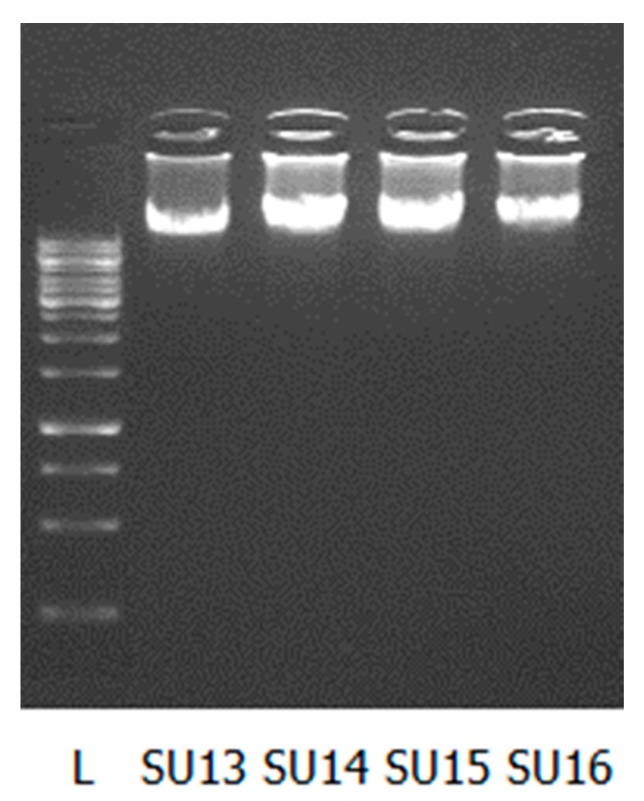
Five µL of extracted DNA was loaded on 1% agarose gel. L—GeneRuler 1 kb DNA Ladder (250–10,000 bp, Thermo Scientific, Roskilde, Denmark); S13–S16 phage DNA.

We successfully extracted DNA from 16 phages active against *Streptococcus uberis.* The DNA quality and quantity are shown in Table 1 as measured with the Nanodrop, Qubit, and by gel electrophoresis (Figure 1). We have also included the results for the *Salmonella* (S112) and *Escherichia coli* (EC11 and EC80) phage DNA extracted using our method for comparison.

The DNA concentration varied from 4.56–287.35 ng/µL. There was no linear correlation observed between the starting concentration of phages and the quantity of DNA extracted. Based on our results, we observed that the optimal concentration of our phages for successful extraction of DNA was above 1.0 × 10^10^ PFU/mL, as no measurable DNA was obtained when titres lower than this were used (e.g., SU11, starting titer = 6.0 × 10^9^PFU/mL, nanodrop concentration = −8.59 ng/µL). The purity of DNA was determined by the A260/280 ratio. Using this measurement, 16/19 DNA samples were of high quality. The integrity of the extracted DNA was determined by gel electrophoresis (Figure 1). An intact band suggests that the DNA is not contaminated with RNA nor degraded.

Despite the low quality scores for a few samples, we were able to prepare libraries and sequence on the Illumina Miseq platform all phage DNA samples. De novo sequence assembly of the trimmed raw reads was performed in the CLC Genomics Workbench v.7 (Qiagen Hilden, Germany) and for each phage, ≥99.85% of reads were assembled into one contig representing the phage genome (Table 2).

## 5. Conclusions

The DNeasy Blood and Tissue kit from Qiagen is commonly used in research laboratories for extraction of bacterial or eukaryotic DNA. Here, we demonstrate that with some modifications it can also be used for purification of phage DNA that can be sequenced on the Illumina MiSeq platform.

Limitations to the technique. The quality and concentration obtained in this study was sufficient for sequencing on the Illumina Miseq platform. However, this DNA might not be of sufficient quality for use on other sequencing platforms such as non-amplification, long read technology e.g., PacBio [9] or Oxford Nanopore [10]. Furthermore, we only extracted DNA from double stranded DNA (dsDNA) phages and it is unknown if this method would work on single-stranded DNA (ssDNA) or RNA viruses.

Method tried on other types of phages. Our streptococcal phages belonging to the Podoviridae-like family were used in this study. We have also used this method on phages active against *E. coli* and *Salmonella* (Table 1 and Table 2) belonging to the Myoviridae and Siphoviridae families.

## Figures and Tables

**Table 1:** Overview of DNA quality and quantity compared to starting phage concentration.

Phage ID	Starting PFU/mL	Nanodrop (ng/µL)	Qubit (ng/µL)	260/280 ^a^
SU1	2.1 × 10^11^	225.67	216	1.86
SU2	9.0 × 10^10^	81.15	80.8	1.82
SU3	9.0 × 10^10^	66.87	56.4	1.88
SU4	6.0 × 10^10^	26.85	17.5	1.65
SU5	1.6 × 10^11^	268.01	224	1.83
SU6	3.0 × 10^11^	69.41	62.4	1.82
SU7	6.0 × 10^10^	95.73	89.6	1.86
SU8	1.0 × 10^11^	105.76	78.4	1.80
SU9	1.1 × 10^11^	59.35	55.2	1.84
SU10	2.0 × 10^11^	287.35	216	1.84
SU11	1.0 × 10^10^	12.67	8.56	1.81
SU12	1.4 × 10^10^	4.56	1.22	1.57
SU13	2.0 × 10^11^	85.73	67.6	1.82
SU14	1.9 × 10^11^	93.16	80.8	1.84
SU15	1.3 × 10^11^	115.78	91.6	1.83
SU16	1.0 × 10^12^	73.95	59.6	1.88
*S112	1.0 × 10^10^	57.60	52	1.60
*EC11	2.8 × 10^10^	30.91	35	1.80
*EC80	2.0 × 10^11^	33.55	24.8	1.84

^a^ Ratio = ~1.8 is accepted as pure DNA. Lower values might be related to the presence of residues of phenol, guanidine, or other reagents used in DNA extraction or an indication of a very low concentration of DNA. * *Salmonella* and *Escherichia coli* phages.

**Table 2:** Summary of read and assembly quality.

Phage ID	Number of Contigs >1000 bp	Max Contig Length	Min Contig Length	N50	Reads Assembled to Max Contig (%)	Number of Paired Reads	Reads with Phred Score Above 30 (%)
SU1	1	34,770	209	34,770	99.85	575,584	96.93
SU2	3	34,819	200	34,819	99.96	547,242	97.58
SU3	1	34,808	201	34,808	99.86	560,430	96.76
SU4	1	34,819	204	34,819	99.94	422,742	95.65
SU5	1	34,800	98	34,800	99.86	238,880	96.95
SU6	1	34,799	205	34,799	99.95	483,526	96.61
SU7	1	34,784	218	34,784	99.98	391,000	93.43
SU8	1	34,802	206	34,802	99.92	354,100	96.86
SU9	1	34,771	204	34,771	99.85	245,804	96.83
SU10	1	34,873	204	34,873	99.99	266,872	95.73
SU11	2	37,919	214	37,919	99.94	247,626	95.79
SU12	1	34,507	221	34,507	99.99	337,214	95.70
SU13	1	34,804	201	34,804	99.93	304,082	92.69
SU14	1	35,029	200	35,029	99.91	294,802	96.27
SU15	1	34,974	205	34,974	99.98	371,720	94.62
SU16	1	34,819	201	34,819	99.99	552,896	94.31
S112	1	52,664	210	52,664	99.97	100,884	93.28
EC11	1	169,478	213	169,478	99.98	206,448	97.31
EC80	1	52,706	202	52,706	99.96	5902	95.20
